# Gut Microbiota Plasticity Influences the Adaptability of Wild and Domestic Animals in Co-inhabited Areas

**DOI:** 10.3389/fmicb.2020.00125

**Published:** 2020-02-07

**Authors:** Wen Qin, Pengfei Song, Gonghua Lin, YanGan Huang, Lei Wang, Xiaowen Zhou, Shengqing Li, Tongzuo Zhang

**Affiliations:** ^1^Key Laboratory of Adaptation and Evolution of Plateau Biota, Northwest Institute of Plateau Biology, Chinese Academy of Sciences, Xining, China; ^2^College of Life Sciences, University of Chinese Academy of Sciences, Beijing, China; ^3^School of Life Sciences, Jinggangshan University, Ji’an, China; ^4^Lanzhou Zoo, Lanzhou, China; ^5^Qinghai Academy of Animal Science and Veterinary Medicine, Qinghai University, Xining, China; ^6^Qinghai Provincial Key Laboratory of Animal Ecological Genomics, Xining, China

**Keywords:** *Gazella subgutturosa*, *Ovis aries*, gut microbiota diversity, gut microbiota functions, adaptability

## Abstract

Due to the increased economic demand for livestock, the number of livestock is increasing. Because of human interference, the survival of wild animals is threatened in the face of competition, particularly in co-inhabited grazing pastures. This may lead to differences in the adaptability between wild and domestic animals, as well as nutritional deficiencies in wild animals. The gut microbiota is closely associated with host health, nutrition, and adaptability. However, the gut microbiota diversity and functions in domestic and wild animals in co-inhabited areas are unclear. To reveal the adaptability of wild and domestic animals in co-inhabited areas based on gut microbiota, we assessed the gut microbiota diversity. This study was based on the V3–V4 region of 16S rRNA and gut microbiota functions according to the metagenome analysis of fresh fecal samples in wild goitered gazelles (*Gazella subgutturosa*) and domestic sheep (*Ovis aries*) in the Qaidam Basin. The wild and domestic species showed significant differences in alpha- and beta-diversities. Specifically, the alpha-diversity was lower in goitered gazelles. We speculated that the nutritional and habitat status of the goitered gazelles were worse. The gut microbiota functions in the gazelles were enriched in metabolism and cellular processes based on the KEGG database. In summary, we reasoned that gut microbiota can improve the adaptability of goitered gazelles through energy maintenance by the functions of gut microbiota in the face of nutritional deficiencies. These findings highlight the importance of gut microbiota diversity to improve the adaptability of goitered gazelles, laying a foundation for the conservation of wild goitered gazelles. In addition, we further provide management suggestions for domestic sheep in co-inhabited grazing pastures.

## Introduction

Wild and domestic animals that co-inhabit the same regions face similar environmental challenges and may compete for food (Xu et al., 2008a). Due to the increasing demand for livestock, their numbers continue to increase (McDonald et al., 2019) and domestic animals may encroach on wildlife resources, imposing new selection pressures on wild animals, particularly in the grazing pastures (Scasta et al., 2016; Zhao et al., 2019). Monitoring the adaptability of wild and domestic animals in co-inhabited areas is essential to the conservation of wild animals and also benefits the management of domestic animals (Woodcock et al., 2005).

The Qaidam Basin is an inland basin in the northern Qinghai Province, marked by drought, levels of evaporation that exceed precipitation, long cold winters, and large temperature variations. The average annual temperatures range from 1.2 to 4.3°C and the elevation ranges from 2600 to 3000 m (Shi et al., 2005; Wei et al., 2014; Zhong et al., 2014), in which the natural conditions are harsh. In the Qaidam Basin, the composition of the vegetation is simple, with deserta most prevalent. The vegetation coverage is less than 5%. The main plants include *Nitraria tangutorum*, *Sympegma regelii*, *Kalidium foliatum*, and *Salsola collina* (Zhong et al., 2014). Meanwhile, the Qaidam Basin is the main habitat of goitered gazelles in Qinghai Province (Northwest Institute of Plateau Biology, 1989) and the main domestic animals here are sheep. *Gazella subgutturosa* (Güldenstaedt, 1780), also known as goitered gazelles, are inhabitants of deserts and semi-deserts (IUCN SSC Antelope Specialist Group, 2017). Studies on goitered gazelles have focused on feeding habits, behavioral characteristics, and physiological and ecological adaptation (Ostrowski and Joseph, 2006; Ostrowski et al., 2006; Chu et al., 2008; Xu et al., 2008a, b; Blank et al., 2012, 2015). Goitered gazelles adapt to water and food shortages by changing their organ size (Ostrowski et al., 2006), but knowledge of their gut microbiota and its adaptability are sparse. Goitered gazelles in the Qaidam Basin are rarely assessed and studies on the gut microbiota of domestic sheep (*Ovis aries*) have been limited to health and nutritional assessments (Tanca et al., 2017; Thomas et al., 2019). As sheep are an economic species, the main points of interest include health maintenance, disease treatment, and weight-gaining approaches (Houston et al., 2000; Al-Dabeeb, 2005; Supratman et al., 2018). The dietary overlap between wild goitered gazelles and domestic sheep during winter in the Karamely Mountain can reach 0.935, suggesting that the two species are likely to face food competition (Chu et al., 2008). Nuomuhong County in the Qaidam Basin (our sampling area) is an area co-inhabited by wild goitered gazelles and domestic animals. Due to human interference, domestic animals generally feed in high-quality pastures. Hence, wild animals inhabiting the Qaidam Basin may face low food quality coupled with severe cold during the winter months (Li et al., 2013). Compared to domestic sheep, wild goitered gazelles face greater survival challenges and higher competitive pressure in winter on the Qaidam Basin. In this study, the Nuomuhong County in the Qaidam basin was selected as a representative site to study the adaptability of wildlife and domestic animals in co-inhabited areas.

The gut microbiota reflects and regulates the metabolic and immune responses of the host, each of which are a key to host adaptation (Ross et al., 2010; Fischbach and Sonnenburg, 2011; Payne et al., 2012; Drissi et al., 2014; Trompette et al., 2014; Sun et al., 2016; Tanca et al., 2017; Gazzaniga and Kasper, 2018; Huang et al., 2018). In herbivores, the gut microbiota is dominated by *Firmicutes* and *Bacteroides*, the functions of which are related to cellulose digestion (De Filippo et al., 2010; Bergmann et al., 2015; Xue et al., 2016). An array of environmental factors influence the composition and function of the gut microbiota, including diet, host genetics (Zhang et al., 2015; Li et al., 2018; Pereira-Marques et al., 2019), and habitat (Huang et al., 2018). Variations in gut microbiota composition and function are associated with food intake (Claesson et al., 2012; Zhao et al., 2017; Zmora et al., 2018). The gut microbiota are influenced by the digestive system of the host, producing specific metabolites that affect both metabolism and host health (Vrieze et al., 2010; Clemente et al., 2012; Xu et al., 2013). Studies on the Alaskan moose found that a high starch diet led to an abundance of archaea in the rumen (Ishaq and Wright, 2012). Due to similar high-cellulose diets in Yunnan snub-nosed monkeys (*Rhinopithecus bieti*) and cows, the gut microbiota diversity of these species is comparable (Xu et al., 2015). When food is abundant, *Bacteroides thetaiotaomicron* fully utilize glycogen. However, when food polysaccharides are in short supply, *Bacteroides thetaiotaomicron* uses proteins and glycolipids to synthesize polysaccharides (Shen, 2012). It is accepted that host genes influence the diversity and function of the gut microbiota (Khachatryan et al., 2008; Turpin et al., 2016), which can be differentiated according to species (Turpin et al., 2016; Ding et al., 2017; Crespo-Piazuelo et al., 2019; Quan et al., 2019). The colonization of microorganisms from the environment into the animal gut represents a screening process. Environmentally ingested microorganisms can be directly excluded or eliminated due to competition with the gut microbiota (Smith et al., 2015). The gut microbiota composition of fish differs in salt *vs.* freshwater (Sullam et al., 2012). Habitat also significantly impacts the gut microbiota of frog species living in farmlands and forests. Due to different selection pressures, the gut microbiota functions of farmland frogs are more diverse (Huang et al., 2018). Habitat degradation is associated with a loss of alpha diversity (Amato et al., 2013). In cold environments, physiological adaptations occur in mammals and the gut microbiota promotes intestinal regulation and absorption, enhancing food and energy utilization (Gomez De La Torre Canny and Rawls, 2015). The gut microbiota also provides energy through the fermentation of non-digestive carbohydrates to short-chain fatty acids (Tremaroli and Bäckhed, 2012). Moreover, the gut microbiota is conducive to host energy compensation (Amato et al., 2015; Sommer et al., 2016). For example, brown bears enhance their energy compensation during hibernation periods (Sommer et al., 2016).

Previous comparative studies on gut microbiota involved captive and wild populations and focused on health and reintroduction problems (Glad et al., 2010; Cheng et al., 2015; Guan et al., 2017; Li et al., 2017). In general, significant differences exist between the gut microbiota of wild and domestic animals (Scupham et al., 2008; Lyu et al., 2018), because of the variation in habitats or diet (Borbon-Garcia et al., 2017; Li et al., 2017). However, these studies were limited to wild animals (Moeller et al., 2013; Menke et al., 2014; Yang et al., 2016). At present, no comparative studies on the gut microbiota diversities between wild and domestic animals in co-inhabited area of the Qaidam Basin have been performed. It is currently unclear whether wild goitered gazelles are influenced by domestic sheep and what the relationship is between gut microbiota and their adaptability. We speculated that due to disturbances in grazing, the nutritional level and habitat quality of wild goitered gazelles was decreased, leading to changes in the gut microbial diversity and function.

In this study, we collected 33 fresh fecal samples by a non-invasive sampling method in the Qaidam Basin to compare the diversity and function of the gut microbiota between wild goitered gazelles (*Gazella subgutturosa*) and domestic sheep (*Ovis aries*). The diversity of the V3-V4 regions of the 16S rRNA community structure and functions based on metagenome data were analyzed. Our results lay the foundation for the conservation of wild animals and the management of domestic animals.

## Materials and Methods

### Ethics Statement

All experiments, including the sample collection methods, followed the principles of the Ethical Committee for Experimental Animal Welfare of the Northwest Institute of Plateau Biology.

### Sample Collection

**FIGURE 1:**
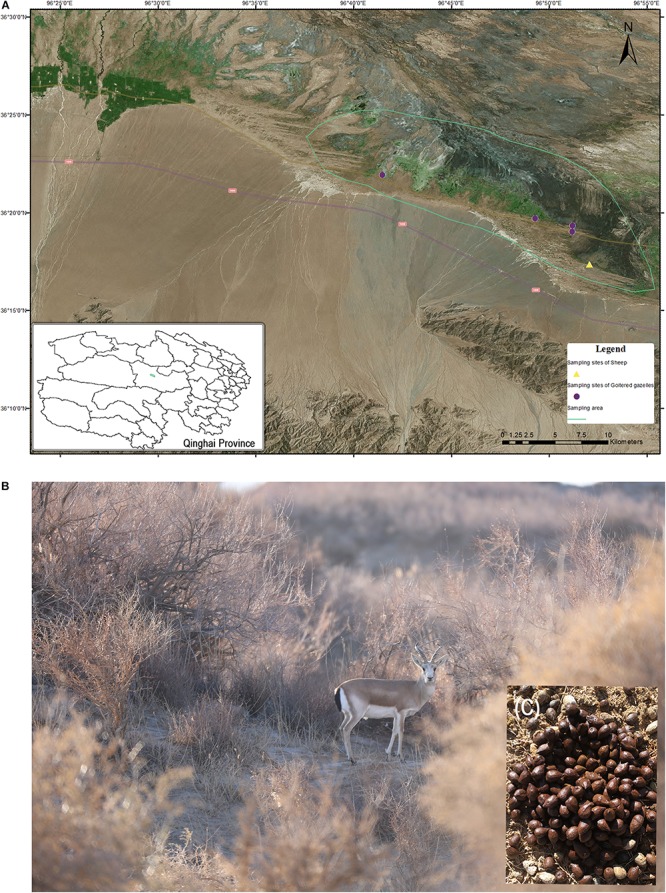
**(A)** Sampling area and sites in Nuomuhong County. **(B)** A male goitered gazelle in its shrub habitat. **(C)** Fresh fecal pellets of a goitered gazelle.

The green solid lines in Figure 1 represent the sampling area in which goitered gazelles and sheep typically forage, though it was not restricted to these areas. According to our investigation, the numbers of goitered gazelles ranged from 150 to 200 (unpublished data) in our sampling area. Goitered gazelles in Nuomuhong County gather together in a regular drinking route (sheep path) in the morning and evening. Their rest shrubs are relatively fixed at night. They typically defecate 1 – 2 times per day and defecation times are concentrated in the mornings and evenings. Fresh fecal material is typically observed near the shrubs where they spend the night. The four sites marked in Figure 1 represent the overnight sites of goitered gazelles, which formed the sampling sites of this study. The sheep feed during the day under the direction of the shepherd and are returned to the sheep pen overnight. The sampling sites of the sheep shown in Figure 1 represent the location of the pen. Samples were collected in the morning prior to the sheep exiting the pen. The sampling areas and sites were geocoded with ArcGIS (V10.5).

The fecal pellets of goitered gazelles were concentrated rather than scattered on the ground, allowing their identification as from individuals not groups. We selected larger, oval samples to ensure they came from adults. Five samples were collected (one per individual) from each sampling site in a single day. Samples were not collected from the same sites on subsequent days to avoid collecting samples from the same individual. The sampling time of the four sites was from December 1 to December 4, 2016. As domestic sheep gather in large groups, only a single sampling point was assessed.

A total of 20 fresh fecal samples from goitered gazelles and 13 fresh fecal samples from domestic sheep were collected. The goitered gazelle samples were labeled from WGS1 to WGS20 and the sheep samples were labeled from WSE1 to WSE13. During sampling, disposable polyethylene (PE) gloves were used to avoid contamination. The collected fecal samples were added to self-sealing bags, numbered, and recorded. The PE gloves were changed upon the collection of subsequent samples. Following collection, all samples were stored at −20°C for a maximum of 1 week. Samples for prolonged storage were stored at −80°C in the Northwest Institution of Plateau Biology. The goitered gazelles and domestic sheep were allowed to naturally defecate for the morning sample collections.

### DNA Extraction, Amplification, and Sequencing

DNA from 33 samples was extracted using the CTAB method and the V3-V4 region of 16S rRNA was amplified using 341F-806R specific primers (341F: 5′-CCTAYGGGRBGCASCAG-3′, 806R: 5′-GGACTACNNGGGTATCTAAT-3′). PCR reactions were performed in a reaction volume of 30 μL and included Phusion^®^ High-Fidelity PCR Master Mix with GC Buffer (New England Biolabs, 15 μL), primers (3 μL), gDNA (10 μL) and H_2_O (2 μL) using the grads PCR instrument (Bio-Rad T100). PCR conditions were as follows: denaturation at 98° for 1 min, 30 cycles of denaturation at 98°C for 10 s, annealing at 50°C for 30 s, extension at 72°C for 30 s, and a final extension at 72°C for 5 min. The PCR products were assessed by 2% agarose gel electrophoresis. The 400 – 450 bp products were gel-purified with GeneJET Gel Extraction Kits (Thermo Scientific).

Illumina TruSeq DNA PCR-Free Library Preparation Kits (Illumina, United States) were used for library sequencing according to the manufacturer’s protocols. Index codes were added to all samples. The Qubit@ 2.0 Fluorometer (Thermo Scientific) and the Agilent Bioanalyzer 2100 systems were used to assess library quality and the 250 bp paired-end reads were obtained after sequencing on the Illumina HiSeq platform.

### Metagenome Sequencing and Annotation

Eight fecal samples (four from goitered gazelles and four from domestic sheep) were randomly selected for metagenome analysis to sequence the total microbial DNA. Qubit was used to quantify the DNA concentrations and the DNA samples were randomly restricted into 350 bp segments using Covaris. Inter-sizes were detected using the Agilent 2100 library and the samples were diluted to 2 ng/uL. The libraries were quantified using Q-PCR and sequenced by Illumina PE150. Reads in the raw data with quality scores ≤ 38, N numbers ≥ 10 bp, and overlap lengths ≥ 15 bp were deleted by Readfq software^1^ (V8). Bowtie2 software was used to avoid host contamination (Karlsson et al., 2012, 2013). The parameters were –end-to-end, –sensitive, and -I 200, -X 400. Clean data were used for subsequent analysis.

Assembly analysis was performed using SOAP *de novo* software (version 2.04) (Luo et al., 2012). The samples were assembled according to K-mer = 55 using parameters of -d 1, -M 3, -R, -u, and -F (Scher et al., 2013; Qin et al., 2014; Brum et al., 2015; Feng et al., 2015). Scaffolds were interrupted from Ns to obtain Scaftigs lacking Ns (Mende et al., 2012; Nielsen et al., 2014; Qin et al., 2014). To acquire unused PE reads, we mapped the clean reads to Scaftigs with Bowtie2 software (Karlsson et al., 2012, 2013) and the parameters were –end-to-end, –sensitive, –I 200, –X 400. The unused reads were mix-assembled based on K-mer = 55 (Qin et al., 2010; Karlsson et al., 2012, 2013; Qin et al., 2014). We used Scaftigs with lengths ≥ 500 bp for subsequent analysis (Karlsson et al., 2013; Li et al., 2014; Nielsen et al., 2014; Zeller et al., 2014; Sunagawa et al., 2015).

ORF (open reading frame) predictions for Scaftigs were produced from mixed assemblies using MetaGeneMark (Zhu et al., 2010; Karlsson et al., 2012; Mende et al., 2012; Li et al., 2014; Qin et al., 2014; Oh et al., 2014). Sequences < 100 nt were discarded (Qin et al., 2010; Nielsen et al., 2014; Zeller et al., 2014; Sunagawa et al., 2015). CD-HIT (Li and Godzik, 2006; Fu et al., 2012) was used to remove redundancies to obtain initial gene catalogs. Cluster default parameters were used to identify 95%, coverage 90%, -c 0.95, -G 0, -aS 0.9, -g 1, and -d 0. The longest sequences were selected as representative. The clean data were mapped to the gene catalog to acquire the numbers of reads in each sample based on Bowtie2 software. The parameters were –end-to-end, –sensitive, -I 200, -X 400. Following gene deletion, the number of reads was ≤2 (Karlsson et al., 2012; Qin et al., 2012). The final gene catalog was obtained following further analysis. According to the read numbers and gene lengths, the relative abundance of the unigenes was calculated.

DIAMONDE software (Buchfink et al., 2015) was used to compared the unigenes, including bacteria, fungi, archaea, and viruses, in the NCBI NR database (Version: 2018.01) (blastp, *e*-value ≤ 1e-5) (Karlsson et al., 2013). We selected data with minimum e-values for further analysis based on the LCA algorithm (Huson et al., 2011). The unigenes were compared to the KEGG database (Kyoto Encyclopedia of Genes and Genomes) with DIAMONDE software to obtain annotation information on functions (blastp, *e*-value ≤ 1e-5) (Li et al., 2014; Feng et al., 2015). Data with the highest scores (one HSP > 60 bits) were selected for subsequent analysis (Qin et al., 2012; Karlsson et al., 2013; Li et al., 2014; Backhed et al., 2015). Relative gene abundances were annotated at the functional level (Karlsson et al., 2012; Li et al., 2014).

### Data Analysis

FLASH (Magoč and Salzberg, 2011) was used to merge paired-end reads to obtain raw Tags. Quality control of the raw Tags was performed with QIIME (Caporaso et al., 2010). Chimeras were removed after comparison of the Tags to the Gold database (Edgar et al., 2011; Haas et al., 2011). Effective Tags were finally obtained.

Effective Tags with ≥ 97% similarities were clustered into the same OTUs (operational taxonomic units) and richness was counted using Uparse software (Edgar, 2013). The highest frequency OTUs were selected as representative and individual singletons were removed with Uparse software (Edgar, 2013). Annotation information was obtained at seven levels (kingdom, phylum, class, order, family, genus, and species) through the comparison of representative OTUs with the SSUrRNA database (Quast et al., 2013) in SILVA (Wang et al., 2007) (threshold: 0.8∼1) using the Mothur method (Schloss et al., 2009). Sequence alignments were performed with MUSCLE software (Edgar, 2004). Alpha- and beta- diversity analyses were performed based on the normalized sample data.

Chao1, Shannon, Simpson, and ACE indices were calculated using Qiime software (Caporaso et al., 2010). The intergroup differences were analyzed with R software^2^ at the alpha-diversity level. At the beta-diversity level, the unweighted and weighted Unifrac distances and UPGMA (Unweighted Pair-group Method with Arithmetic Means) trees were calculated using Qiime software (Caporaso et al., 2010). PCA analysis (packages “ade4” and “ggplot2”) (Dray et al., 2018; Wickham et al., 2019), Anosim analysis (packages “vegan,” anosim function) (Oksanen et al., 2019), heatmap (packages “pheatmap”) (Perry, 2016) and Metastats analysis were performed with R software (packages “optparse”) (Davis, 2019). LefSe (linear discriminant analysis effect size) analysis was performed using LefSe software (Segata et al., 2011). The LDA (linear discriminant analysis) score was 4.

## Results

### Gut Microbiota Profiles

**FIGURE 2:**
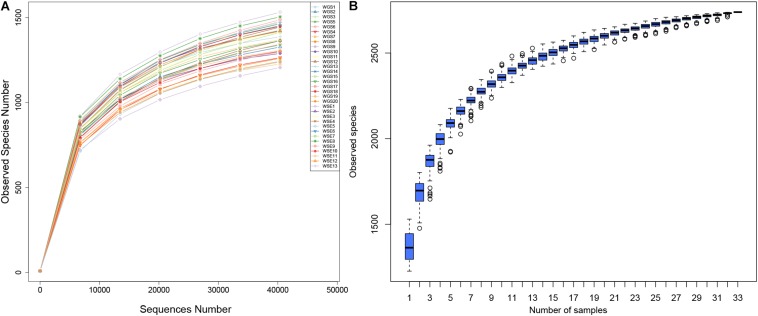
**(A)** Rarefaction Curves. *X*-axis: number of randomly selected sequences in the samples; *y*-axis: number of OTUs based on the sequences. Individual samples are represented by different colors. **(B)** Species accumulation boxplots. *X*-axis: sample size; *y*-axis: number of OTUs after sampling.

According to 16S rRNA data, we identified 2,626,321 reads, 2,358,917 of which were combined with an average length of 410.15 bp (Supplementary Table A). A total of 2,107,371 qualified reads were produced, including 63,860 reads per sample. The qualified reads ranged from 45,828 to 72,205 per sample. The average length of the reads was 409.7 bp, with Q20 ≥ 98% and Q30 ≥ 96% (Supplementary Table B). Both the rarefaction curves and species accumulation plots indicated a relationship between sequencing depth and OTU numbers. All rarefaction curves (Figure 2A) were smooth, indicating a sufficient sequence depth with a very low possibility of discovering new OTUs. The species accumulation boxplots (Figure 2B) tended to be smooth, indicating sufficient sequencing depth. The possibility of new OTUs did not significantly increase with larger sampling size.

**FIGURE 3:**
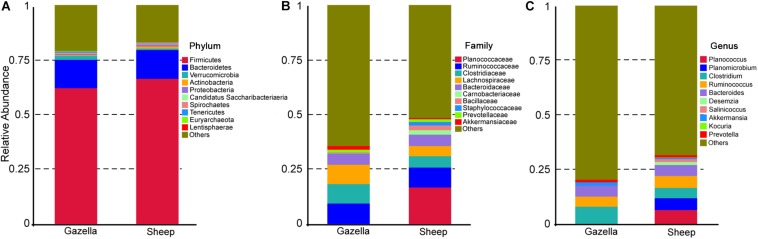
**(A)** Phylum level: top 10 phyla in the gut microbiota of goitered gazelles and domestic sheep. **(B)** Family level, top 10 families in the gut microbiota of goitered gazelles and domestic sheep. **(C)** Genus level, top 10 genera in the gut microbiota of goitered gazelles and domestic sheep.

At the OTU level, according to 97% sequence-similarity thresholds, 2205 OTUs were shared by the goitered gazelles and domestic sheep. A total of 317 OTUs were unique to goitered gazelles, whereas 213 OTUs were unique to domestic sheep. This suggested that the composition of the two species is comparable at the OTU level. The gut microbiota of the goitered gazelles was classified into 25 phyla, 130 families and 246 genera (including unclassified entries). The gut microbiota of the sheep was divided into 21 phyla, 112 families, and 229 genera (including unclassified entries). The relative abundance of the top 10 phyla, top 10 families, and top 10 genera is shown in Figure 3.

### Gut Microbiota Composition

**FIGURE 4:**
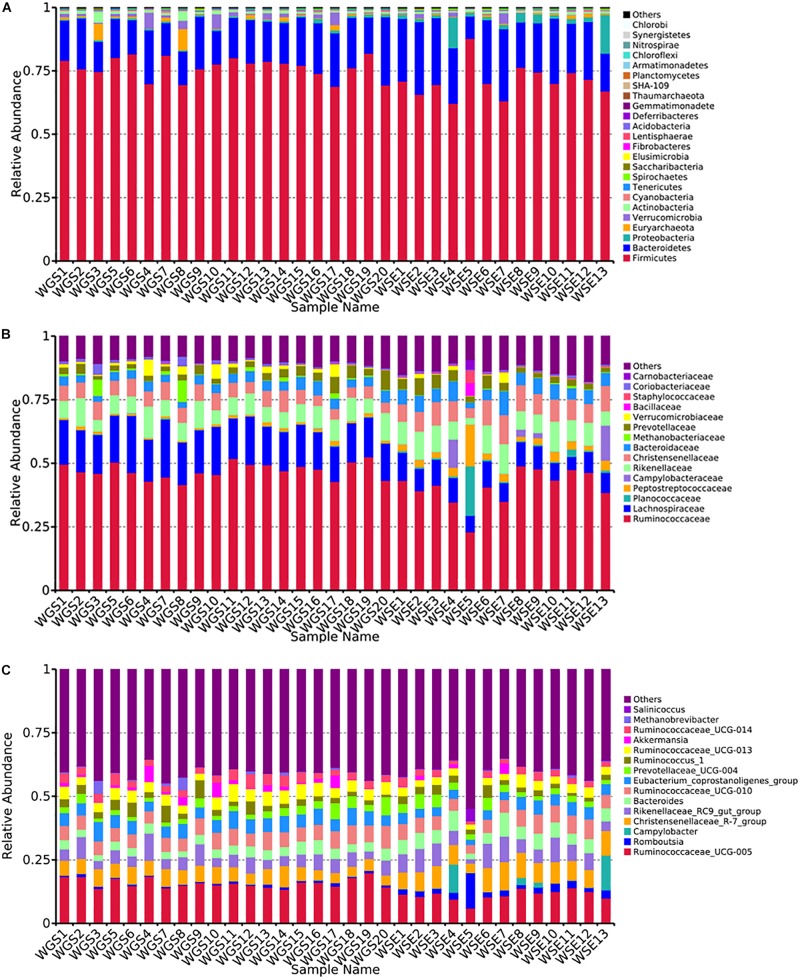
**(A)** Phylum level: top 25 phyla in the gut microbiota samples. **(B)** Family level: top 15 families in the gut microbiota. **(C)** Genus level: top 15 genera in the gut microbiota samples.

At the phylum level, *Firmicutes* (76.40% ± 0.93%; 71.03% ± 1.83%) and *Bacteroides* (17.17% ± 0.85%; 21.84% ± 1.58%) were the core phyla (relative abundance ≥ 1%) in both the goitered gazelles and sheep. These results were consistent with other previous studies (Bergmann et al., 2015; Xue et al., 2016; Tanca et al., 2017). The *Firmicutes/Bacteroides* ratio in goitered gazelles was 4.670 ± 1.091 and 3.686 ± 2.012 in sheep. The bacteria from the two phyla are related to cellulose and carbohydrate digestion (De Filippo et al., 2010; Li et al., 2017). According to Metastats analysis, the relative abundance of *Firmicutes* in the goitered gazelles was significantly higher than that in domestic sheep, whereas the relative abundance of *Bacteroides* was significantly lower (*P* < 0.05). *Thaumarchaeota*, *Synergistetes*, *Chlorobi*, and *TM6* were only identified in goitered gazelles. The relative abundance of each sample at the phylum level is shown in Figure 4A.

We identified 10 core families (including unclassified families) shared by both species. The top three families in terms of relative abundance were *Ruminococcaceae* (47.14% ± 0.70%, 40.71% ± 1.96%), *Lachnospiraceae* (16.92% ± 0.53%, 8.50% ± 0.47%), and *Rikenellaceae* (7.83% ± 0.47%, 8.64% ± 0.75%), all shared by both species. The relative abundance of *Ruminococcaceae* and *Lachnospiraceae* in goitered gazelles was significantly higher than that in domestic sheep (*P* < 0.05), whereas the relative abundance of *Enterobacteriaceae* was lower in gazelles (*P* < 0.01). In the 10 core families, six showed significance (*P* < 0.05) based on Metastats analysis. These included *Ruminococcaceae*, *Lachnospiraceae*, *Christensenellaceae* (5.79% ± 0.27%, 8.42% ± 0.53%), *Bacteroidaceae* (3.69% ± 0.19%, 5.65% ± 0.48%), *Peptostreptococcaceae* (1.03% ± 0.09%, 3.87% ± 1.07%), and *Peptococcaceae* (1.01% ± 0.08%, 1.32% ± 0.10%) in goitered gazelles and sheep, respectively. The relative abundance of each sample at the family level is shown in Figure 4B.

At the genus level, 13 core genera (including unclassified genera) were shared by the goitered gazelles and the sheep. According to Metastats analysis, only eight genera showed significant differences (*P* < 0.05), including *Ruminococcaceae_UCG-005* (15.93% ± 0.42%, 11.17% ± 0.58%), *Christensenellaceae_R-7_group* (5.66% ± 0.27%, 8.10% ± 0.51%), *Eubacterium_coprostanoligenes_group* (5.39% ± 0.22%, 4.11% ± 0.27%), *Ruminococcaceae_UCG-013* (4.62% ± 0.22%, 3.28% ± 0.23%), *Bacteroides* (3.69% ± 0.19%, 5.65% ± 0.48%), *Ruminococcaceae_UCG-014* (3.26% ± 0.22%, 2.28% ± 0.19%), *Tyzzerella_4* (2.30% ± 0.08%, 1.12% ± 0.07%), and *Alistipes* (1.64% ± 0.15% 1.16% ± 0.07%) in goitered gazelles and sheep, respectively. The predominant bacteria in goitered gazelles was consistent with sika deer and takin and included *Ruminococcaceae_UCG-005* and *Ruminococcaceae_UCG-010* that are related to cellulose degradation (Chen et al., 2017; Guan et al., 2017). *Prevotella* is a common genus in the gut microbiota of herbivores (Xue et al., 2016; Wang et al., 2017) but was not identified in either the goitered gazelles or the sheep. The relative abundance of each sample at the genus level is shown in Figure 4C.

Potentially pathogenic bacteria also colonized the gastrointestinal tract of both species. The relative abundance of *Campylobacter*, *Helicobacter*, and *Shigella* in the sheep was significantly higher than that of the goitered gazelles. *Campylobacter* is associated with inflammatory bowel disease and sheep abortions (Skirrow, 1994; Gradel et al., 2009). *Helicobacter* is related to peptic ulceration and gastric neoplasia (Blaser and Atherton, 2004). *Shigella* is related to bacterial dysentery (Seekatz et al., 2013). The relative abundance of *streptococcus* in the goitered gazelles was significantly higher than that in the sheep. Some streptococcal species are pathogenic and cause diseases such as pharyngitis, necrotizing fasciitis, and streptococcal toxic shock syndrome (Athey et al., 2016).

### Intragroup and Intergroup Differences in Gut Microbiota Structures

**FIGURE 5:**
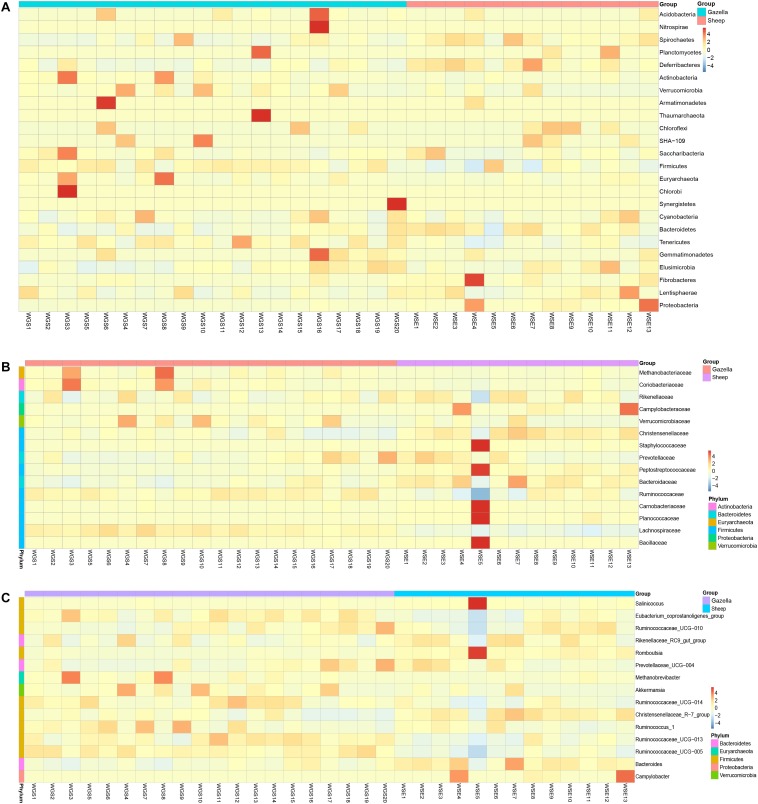
Heatmap of the top 15 bacteria in all samples **(A)** at the phylum level, **(B)** family level, **(C)** and genus level.

From assessment of the gut microbiota structures (Figures 3, 4) and the heatmap (Figure 5), the composition of all samples was similar. The composition of WSE5 did differ, but this sample was not removed.

**FIGURE 6:**
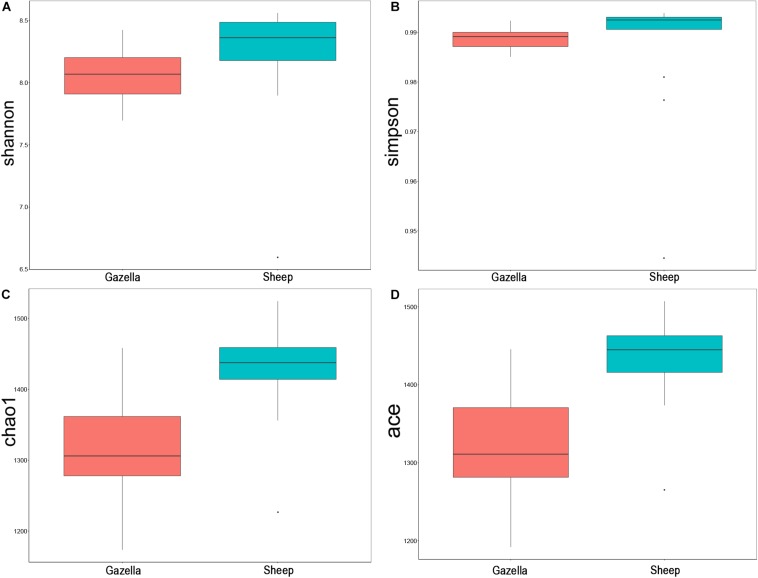
Comparison of alpha-diversity indexes between goitered gazelles and sheep based on the **(A)** Shannon, **(B)** Simpson, **(C)** Chao 1, and **(D)** ACE indices.

**FIGURE 7:**
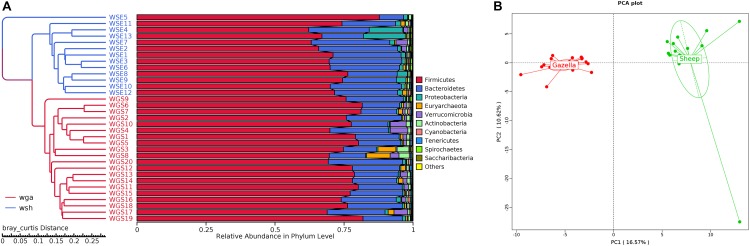
**(A)** Bray–Curtis UPGMA tree. The samples are labeled in different colors. **(B)**: Cluster analysis by PCA (principal component analysis).

**FIGURE 8:**
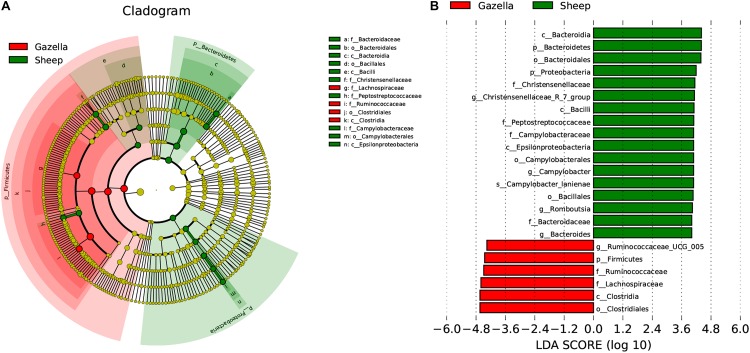
Lefse analysis of the gut microbiota in goitered gazelles and domestic sheep. **(A)** Cladogram of gut microbiota communities. **(B)** Biomarker genes and their LDA scores (LDA score = 4).

At the alpha-diversity level, according to Wilcoxon tests, the Shannon index (goitered gazelle = 8.06; sheep = 8.36; *P* = 0.0297), Simpson index (goitered gazelle = 0.989; sheep = 0.992; *P* = 0.0219), Chao 1 index (goitered gazelle = 1306; sheep = 1438; *P* = 0.00064), and ACE index (goitered gazelle = 1311; sheep = 1445; *P* = 0.000071) (Figure 6), the gut microbiota in sheep was more diverse than that in goitered gazelles. From the UPGMA tree (Bray–Curtis), the gut microbiota of goitered gazelles and sheep clustered into two categories that were distinctly separated (Figure 7A). At the beta-diversity level, PCA (principal component analysis) (Figure 7B) showed clear differences between the groups, which was confirmed by Anosim analysis (*R* = 0.867, *P* = 0.001), indicating significant differences between goitered gazelles and sheep. The intergroup distances were greater than the intragroup differences. According to Lefse analysis (Figure 8), 23 biomarkers were identified (LDA score: 4). The relative abundance of *Bacteroidetes*, *Proteobacteria*, *Christensenellaceae*, *Peptostreptococcaceae*, *Bacteroides* and *Romboutsia* was significantly higher in sheep, whereas *Firmicutes*, *Ruminococcaceae*, and *Ruminococcaceae_UCG-005* were significantly higher in goitered gazelles.

### Comparative Analysis of Metagenome Functions and Contributing Bacteria

We obtained a total of 401,727,108 reads, 60,259.07 M of clean data. Q30 and Q20 were above 96 and 90%, respectively, in the clean data. We obtained 1,800,923 ORFs with an average length of 596.86 bp based on the metagenomic analysis.

The functions of gut microbiota in the goitered gazelles and sheep were mainly enriched in “Metabolism” and “Cellular Processes” (*P* < 0.05). Forty-nine functions were significantly enriched in the goitered gazelles (*P* < 0.05) (Supplementary Table C), mainly regarding “Carbohydrate and amino acid metabolism.” Moreover, the relative abundance of 49 functions in the goitered gazelles was greater than that of the sheep. The top six were “Starch and sucrose metabolism” (ko00500), “Cysteine and methionine metabolism” (ko00270), “Galactose metabolism” (ko00052), “Peptidoglycan biosynthesis” (ko00550), “Oxidative phosphorylation” (ko00190), and “Phenylalanine, tyrosine and tryptophan biosynthesis” (ko00400). The gut microbiota of the goitered gazelles was significantly enriched in energy metabolism. Six significant differences associated with Cellular Processes were observed between the goitered gazelles and the sheep (Supplementary Table D), including Biofilm formation – *Pseudomonas aeruginosa*, Ferroptosis, Cell cycle – *Caulobacter*, Biofilm formation – *Vibrio cholerae*, Autophagy – yeast, and Peroxisomes. The relative abundances in the goitered gazelles were also higher than those in the sheep.

**FIGURE 9:**
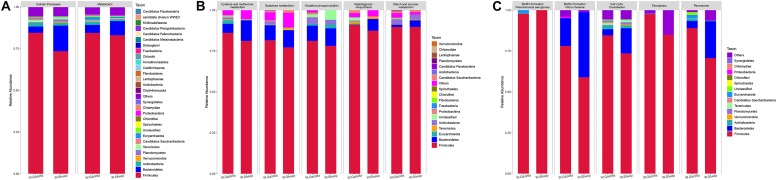
**(A)** Relative abundance of functional genes based on KEGG database and their contributing bacteria between goitered gazelle and domestic sheep with the same distribution at first level; **(B)** the relative abundance of contributing bacteria of top 5 functions showing significant differences between goitered gazelle and domestic sheep with the same distribution according to KEGG database at third level in Metabolism; **(C)** the relative abundance of contributing bacteria of top 5 functions showing significant differences at third level in Cellular Processes.

According to species analysis with R software (Figure 9), *Firmicutes* and *Bacteroidetes* were the dominant phyla that contributed to “Metabolism” in both the goitered gazelles and the sheep. The remaining phyla that contributed to “Metabolism” were *Actinobacteria*, *Euryarchaeota*, *Candidatus Saccharibacteria*, *Tenericutes*, *Proteobacteria*, *Synergistetes*, and *Planctomycetes*. *Firmicutes* and *Bacteroidetes* were the dominant phyla contributing to Cellular Processes, followed by *Actinobacteria*, *Tenericutes*, *Candidatus Saccharibacteria*, *Euryarchaeota*, and *Planctomycetes*.

## Discussion

### Main Factors in Alpha- and Beta- Diversity Between the Two Species

Differences in food composition are the major determinates of gut microbiota diversity (Ding et al., 2017; Ren et al., 2017). The food composition of goitered gazelles and sheep is different significantly (Chu et al., 2008; Xu et al., 2008a). The host genome dictates the nature of the gut microbiota (Turpin et al., 2016; Crespo-Piazuelo et al., 2019), explaining its species variation (Ding et al., 2017; Li et al., 2017; Quan et al., 2019). Goitered gazelles and sheep belong to *Gazella* and *Ovis* respectively, the relationship of which is distant (Chen and Jiang, 2013). We, therefore, speculated that significant differences in the gut microbiota structures between goitered gazelles and sheep would exist due to dietary and host genetic factors.

In the winter, the dietary niches of the goitered gazelles and sheep overlap, indicating food competition between the two species (Chu et al., 2008). Due to human intervention, the food quality of goitered gazelles is poorer than that of sheep. Sheep have a smaller range of activities and consume larger amounts of grass of high nutritional quality. In contrast, goitered gazelles have a wider forage range and often consume plants of poor nutritional quality (Xu et al., 2008a). The gut microbiota diversity is closely influenced by host-specific feeding ecology (De Filippo et al., 2010; Zmora et al., 2018). In winter, sheep consume higher levels of *Stipa* and *Ceratoides*, whereas goitered gazelles consume more *Haloxylon ammodendron*, *Phragmites australis*, *Nitratia SPP*, and *Reaumuria soongorica* (Chu et al., 2008; Xu et al., 2008a). *Stipa* has both high palatability and nutritional value, with a high content of crude protein, crude fat, and nitrogen-free extract, and low levels of crude fiber (Lu, 2016). The levels of crude protein, crude fat, and nitrogen-free extracts of *Haloxylon ammodendron*, *Phragmites australis*, and *Nitratia SPP* were lower than that of *Stipa*, whereas the content of crude fiber was higher than that of *Stipa* (Gao and Ji, 1996; Wu et al., 2017; Wang and Wu, 2018). Crude protein is the main nutrient of herbage. Crude fat and nitrogen-free extracts provide heat and energy (Lu, 2016). We speculate that, although the diversity and evenness index of food consumed by the goitered gazelles were higher than those of the sheep (Chu et al., 2008), due to the differences in nutritional structure, the gut microbiota diversity was lower than that of sheep (Hekmatdoost et al., 2008) and high levels of *Ceratoides* in sheep contribute to the increase in bacterial diversity in rumens (Yang et al., 2019).

### The Adaptability Strategies of Sheep in Winter

The dietary composition observed in sheep seems to provide them an advantage to deal with harsh winter situations over the diet observed in goitered gazelles. The relative abundance of *Peptococcaceae*, *Christensenellaceae*, and *Bacteroides* in the domestic sheep was higher than that in the goitered gazelles and these bacteria improve the utilization of food and energy. *Peptococcaceae* is related to butyrate synthesis, through which colonocytes obtain their energy requirements (Nam et al., 2018) and increase their energy intake through the degradation of cellulose (Li et al., 2015). *Bacteroidetes* can improve both nutrient efficiency and host immunity by degrading carbohydrates and maintaining intestinal balance (Li et al., 2017; Zhang et al., 2018).

### The Adaptability Strategies of Goitered Gazelles in Winter

The relative abundance of gut microbiota related to cellulose degradation in the goitered gazelles was higher than that of the sheep. *Firmicutes* can degrade cellulose into volatile fatty acids to provide energy for the host, whereas *Bacteroidetes* degrade carbohydrates and proteins to improve the utilization rates of the host (Li et al., 2017). *Ruminococcaceae* are rich in cellulase genes (Amato et al., 2015), which enables goitered gazelles to digest high-fiber plants, such as *Haloxylon ammodendron* and *Phragmites australis*. A high *Firmicutes/Bacteroides* ratio was relevant to energy extraction from the diet (Ma et al., 2019). The higher relative abundance of *Lachnospiraceae* in the gut microbiota of goitered gazelles contributes to homeostatic balance, butyrate production, and pathogen elimination (McLellan et al., 2013). The genomes of a single species of *Alistipes* were enriched in carbohydrate, amino acid, and energy conversion pathways (Preidis et al., 2015). The gut microbiota of the goitered gazelles allows them to obtain energy through the improved utilization of food, whereas energy compensation strategies permit survival in harsh winter environments. The alpha-diversity is higher in undisturbed areas (Barelli et al., 2015). The alpha-diversity of the goitered gazelles was less than that of the sheep, which indicates that the quality or areas of habitats may be decreased in the goitered gazelles. We speculate that, although the survival conditions of the goitered gazelles were worse than those of the sheep, they can be replenished by the gut microbiota.

Digestive strategies are also closely related to the gut microbiota (Singh et al., 2011), with different digestive strategies permitting adaptations for foraging (Clauss et al., 2003). The common bacteria in herbivores are *Firmicutes*, *Bacteriodetes*, and *Proteobacteria* (Cersosimo et al., 2015; Chen et al., 2017; Guan et al., 2017). The higher resistance of wild animals may be associated with physiological adaptations and metabolites produced by the gut microbiota. Wild herbivores more efficiently process foods low in protein and high in fiber (Nelson et al., 2003). Presently, digestive strategies are known to impact both the gut microbiota and host adaptability, but their specific mechanisms require further elucidation in goitered gazelles.

The functions of the gut microbiota demonstrate how goitered gazelles improve their adaptability by enhancing the functions of “Metabolism” and “Cellular Processes” to account for a loss of food quality in winter compared to sheep. The sugar metabolism in goitered gazelles is significantly higher than that of sheep. Sugar metabolism provides energy for the host and is an important energy source (Chen, 2019) that benefits the survival of goitered gazelles. Drought-resistant plants, such as *Reaumuria soongorica*, are consumed by goitered gazelles and are rich in polysaccharides and polyphenols (Wang et al., 2011), which also improve the metabolism of gut microbiota.

The Qaidam Basin is the main habitat of wild goitered gazelles in China. Goitered gazelles are an important wild ungulate in the Qaidam Basin, the stability of which is vital to the biodiversity and ecosystem of the area. Understanding the living conditions of goitered gazelles and their adaptive strategies forms the foundation of their conservation. With developing economic and breeding technologies, livestock numbers are increasing, threatening the survival of wild animals, particularly in co-inhabited areas. In this study, food competition between wild goitered gazelles and domestic sheep was demonstrated and due to human interference, the food quality of the wild goitered gazelles was poorer than that of domestic sheep. The differences in the composition of gut microbiota reflected that wild goitered gazelles were disadvantaged by domestic sheep, but that the gut microbiota of gazelles benefited host adaption via compensatory strategies to enhance host adaptability, improving the utilization rates of food and metabolic levels. This indicated that the gut microbiota benefit host adaptability. However, the side effects of these compensatory mechanisms for the host require further assessment.

## Conclusion

In this study, the composition and function of gut microbiota between wild goitered gazelles and domestic sheep in the Qaidam Basin were compared. We further assessed the adaptability strategies of goitered gazelles using non-invasive methods, which lays a foundation for the conservation of wild goitered gazelles and the management of domestic sheep. With the development of sequencing technologies, variations in the gut microbiota were identified, which highlighted the conflicts between wild and domestic animals in co-inhabited areas. We evaluated adaptability based solely on the gut microbiota, for which goitered gazelles in the Qaidam Basin have not been systematically studied. In future studies, host gene structure and diet should be analyzed in larger sample sizes.

Since goitered gazelles typically rest in shrub areas overnight and follow fixed movement routes during the day, the protection of their habitats should be prioritized. The land use mode of the Qaidam Basin should be planned and pastures with good food quality must be preserved, reducing grazing activities in co-inhabited areas to minimize disruption. Winter is the most severe period for the survival of goitered gazelles and food competition between wild goitered gazelles and domestic sheep heightens this problem. To maintain the survival of goitered gazelles, the animals should be fed green silage with a high protein content during the winter. We further suggest that variations in dietary habits and the gut microbiota in goitered gazelles and sheep should be assessed through feces collection. However, domestic sheep, a major economic source, should be allowed to graze. Suitable pastures for domestic animals should be identified to reduce conflicts with wild animals.

## Data Availability Statement

The datasets generated for this study can be found in NCBI GenBank, accession numbers are PRJNA560474 (https://www.ncbi.nlm.nih.gov/bioproject/?term=PRJNA560474) and PRJNA560663 (https://www.ncbi.nlm.nih.gov/bioproject/?term= PRJNA560663).

## Ethics Statement

The animal study was reviewed and approved by the Ethical Committee for Experimental Animals’ Welfare of Northwest Institute of Plateau Biology. Written informed consent for participation was not obtained from the owners because we collect animal feces by non-damage method, there is no harm to animals.

## Author Contributions

WQ, PS, and XZ wrote the manuscript. GL, SL, and PS analyzed the data. WQ, LW, and YH contributed to sampling and laboratory experiments. TZ designed the study.

## Conflict of Interest

The authors declare that the research was conducted in the absence of any commercial or financial relationships that could be construed as a potential conflict of interest.

## Footnotes

1 The git hub address is https://github.com/cjfields/readfq.

2 The website address is https://www.r-project.org/.

## References

[B1] Al-Dabeeb S. N. (2005). Effect of feeding low quality date palm on growth performance and apparent digestion coefficients in fattening Najdi sheep. *Small Rumin. Res.* 57 37–42. 10.1016/j.smallrumres.2004.05.002

[B2] Amato K. R. Leigh S. R. Kent A. Mackie R. I. Yeoman C. J. Stumpf R. M. (2015). The gut microbiota appears to compensate for seasonal diet variation in the wild black howler monkey (*Alouatta pigra*). *Microb. Ecol.* 69 434–443. 10.1007/s00248-014-0554-7 25524570

[B3] Amato K. R. Yeoman C. J. Kent A. Righini N. Carbonero F. Estrada A. (2013). Habitat degradation impacts black howler monkey (*Alouatta pigra*) gastrointestinal microbiomes. *ISME J.* 7 1344–1353. 10.1038/ismej.2013.16 23486247 PMC3695285

[B4] Athey T. B. Teatero S. Sieswerda L. E. Gubbay J. B. Marchand-Austin A. Li A. (2016). High incidence of invasive group A *Streptococcus* disease caused by strains of uncommon emm types in Thunder Bay, Ontario, Canada. *J. Clin. Microbiol.* 54 83–92. 10.1128/Jcm.02201-15 26491184 PMC4702752

[B5] Backhed F. Roswall J. Peng Y. Feng Q. Jia H. Kovatcheva-Datchary P. (2015). Dynamics and stabilization of the human gut microbiome during the first year of life. *Cell Host Microbe* 17 690–703. 10.1016/j.chom.2015.04.004 25974306

[B6] Barelli C. Albanese D. Donati C. Pindo M. Dallago C. Rovero F. (2015). Habitat fragmentation is associated to gut microbiota diversity of an endangered primate: implications for conservation. *Sci. Rep.* 5:14862. 10.1038/srep14862 26445280 PMC4595646

[B7] Bergmann G. T. Craine J. M. Robeson M. S. II Fierer N. (2015). Seasonal shifts in diet and gut microbiota of the American bison (*Bison bison*). *PLoS One* 10:e0142409. 10.1371/journal.pone.0142409 26562019 PMC4642958

[B8] Blank D. Ruckstuhl K. Yang W. (2012). Influence of population density on group sizes in goitered gazelle (*Gazella subgutturosa* Guld., 1780). *Eur. J. Wildl. Res.* 58 981–989. 10.1007/s10344-012-0641-3

[B9] Blank D. A. Ruckstuhl K. Yang W. (2015). Seasonal dynamics of agonistic displays in territorial and non-territorial males of goitered *Gazelle*. *Zoology* 118 63–68. 10.1016/j.zool.2014.08.004 25435489

[B10] Blaser M. J. Atherton J. C. (2004). *Helicobacter pylori* persistence: biology and disease. *J. Clin. Invest.* 113 321–333. 10.1172/Jci20925 14755326 PMC324548

[B11] Borbon-Garcia A. Reyes A. Vives-Florez M. Caballero S. (2017). Captivity shapes the gut microbiota of andean bears: insights into health surveillance. *Front. Microbiol.* 8:1316. 10.3389/fmicb.2017.01316 28751883 PMC5507997

[B12] Brum J. R. Ignacio-Espinoza J. C. Roux S. Doulcier G. Acinas S. G. Alberti A. (2015). Patterns and ecological drivers of ocean viral communities. *Science* 348:1261498. 10.1126/science.1261498 25999515

[B13] Buchfink B. Xie C. Huson D. H. (2015). Fast and sensitive protein alignment using diamond. *Nat. Methods* 12 59–60. 10.1038/nmeth.3176 25402007

[B14] Caporaso J. G. Kuczynski J. Stombaugh J. Bittinger K. Bushman F. D. Costello E. K. (2010). Qiime allows analysis of high-throughput community sequencing data. *Nat. Methods* 7 335–336. 10.1038/nmeth.f.303 20383131 PMC3156573

[B15] Cersosimo L. M. Lachance H. St-Pierre B. Van Hoven W. Wright A. D. (2015). Examination of the rumen bacteria and methanogenic archaea of wild impalas (*Aepyceros melampus* melampus) from Pongola, South Africa. *Microb. Ecol.* 69 577–585. 10.1007/s00248-014-0521-3 25351144

[B16] Chen J. Jiang Z. (2013). Discrimination of *Saiga* antelope horn from substitutes in“Lingyangjiao”markets by genetic identification technology. *Modern Chin. Med.* 15 548–551. 10.13313/j.issn.1673-4890.2013.07.016

[B17] Chen J. Zhang H. Wu X. Shang S. Yan J. Chen Y. (2017). Characterization of the gut microbiota in the golden takin (*Budorcas taxicolor* bedfordi). *AMB Express* 7:81. 10.1186/s13568-017-0374-5 28413853 PMC5392452

[B18] Chen L. (2019). Research on the mechanism of exercise intervention on glucose metabolism in the skeletal system. *Chin. J. Osteoporos.* 25 1185–1191.

[B19] Cheng Y. Fox S. Pemberton D. Hogg C. Papenfuss A. T. Belov K. (2015). The Tasmanian devil microbiome-implications for conservation and management. *Microbiome* 3:76. 10.1186/s40168-015-0143-0 26689946 PMC4687321

[B20] Chu H. Jiang Z. Lan W. Wang C. Tao Y. Jiang F. (2008). Dietary overlap among kulan *Equus hemionus*, goitered gazelle *Gazella subgutturosa* and livestock. *Acta Zool. Sin.* 54 941–954.

[B21] Claesson M. J. Jeffery I. B. Conde S. Power S. E. O’connor E. M. Cusack S. (2012). Gut microbiota composition correlates with diet and health in the elderly. *Nature* 488 178–184. 10.1038/nature11319 22797518

[B22] Clauss M. Lechner-Doll M. Streich W. J. R. (2003). Ruminant diversification as an adaptation to the physicomechanical characteristics of forage. A reevaluation of an old debate and a new hypothesis. *Oikos* 102 253–262. 10.1034/j.1600-0706.2003.12406.x

[B23] Clemente J. C. Ursell L. K. Parfrey L. W. Knight R. (2012). The impact of the gut microbiota on human health: an integrative view. *Cell* 148 1258–1270. 10.1016/j.cell.2012.01.035 22424233 PMC5050011

[B24] Crespo-Piazuelo D. Migura-Garcia L. Estelle J. Criado-Mesas L. Revilla M. Castello A. (2019). Association between the pig genome and its gut microbiota composition. *Sci. Rep.* 9:8791. 10.1038/s41598-019-45066-6 31217427 PMC6584621

[B25] Davis T. L. (2019). *optparse: Command Line Option Parser. R package version 1.6.4.*

[B26] De Filippo C. Cavalieri D. Di Paola M. Ramazzotti M. Poullet J. B. Massart S. (2010). Impact of diet in shaping gut microbiota revealed by a comparative study in children from Europe and rural Africa. *Proc. Natl. Acad. Sci. U.S.A.* 107 14691–14696. 10.1073/pnas.1005963107 20679230 PMC2930426

[B27] Ding J. Dai R. Yang L. He C. Xu K. Liu S. (2017). Inheritance and establishment of gut microbiota in chickens. *Front. Microbiol.* 8:1967. 10.3389/fmicb.2017.01967 29067020 PMC5641346

[B28] Dray S. Dufour A.-B. Thioulouse J. Jombart T. Pavoine S. Lobry J. R. (2018). *Analysis of Ecological Data: Exploratory and Euclidean Methods in Environmental Sciences Version 1.7-2.*

[B29] Drissi F. Merhej V. Angelakis E. El Kaoutari A. Carriere F. Henrissat B. (2014). Comparative genomics analysis of *Lactobacillus* species associated with weight gain or weight protection. *Nutr. Diabetes* 4:e109. 10.1038/nutd.2014.6 24567124 PMC3940830

[B30] Edgar R. C. (2004). Muscle: multiple sequence alignment with high accuracy and high throughput. *Nucleic Acids Res.* 32 1792–1797. 10.1093/nar/gkh340 15034147 PMC390337

[B31] Edgar R. C. (2013). Uparse: highly accurate Otu sequences from microbial amplicon reads. *Nat. Methods* 10 996–998. 10.1038/nmeth.2604 23955772

[B32] Edgar R. C. Haas B. J. Clemente J. C. Quince C. Knight R. (2011). Uchime improves sensitivity and speed of chimera detection. *Bioinformatics* 27 2194–2200. 10.1093/bioinformatics/btr381 21700674 PMC3150044

[B33] Feng Q. Liang S. Jia H. Stadlmayr A. Tang L. Lan Z. (2015). Gut microbiome development along the colorectal adenoma-carcinoma sequence. *Nat. Commun.* 6:6528. 10.1038/ncomms7528 25758642

[B34] Fischbach M. A. Sonnenburg J. L. (2011). Eating for two: how metabolism establishes interspecies interactions in the gut. *Cell Host Microbe* 10 336–347. 10.1016/j.chom.2011.10.002 22018234 PMC3225337

[B35] Fu L. Niu B. Zhu Z. Wu S. Li W. (2012). Cd-Hit: accelerated for clustering the next-generation sequencing data. *Bioinformatics* 28 3150–3152. 10.1093/bioinformatics/bts565 23060610 PMC3516142

[B36] Gao W. Ji L. (1996). An analysis of the nutrient composition of five species of sandy plants growing in west Inner Mongonia. *J. Argric. Anim. Husb.* 17 23–28.

[B37] Gazzaniga F. S. Kasper D. L. (2018). Wild gut microbiota protects from disease. *Cell Res.* 28 135–136. 10.1038/cr.2017.150 29192675 PMC5799813

[B38] Glad T. Kristiansen V. F. Nielsen K. M. Brusetti L. Wright A. D. Sundset M. A. (2010). Ecological characterisation of the colonic microbiota in arctic and sub-arctic seals. *Microb. Ecol.* 60 320–330. 10.1007/s00248-010-9690-x 20523986

[B39] Gomez De La Torre Canny S. Rawls J. F. (2015). Baby, it’s cold outside: host-microbiota relationships drive temperature adaptations. *Cell Host Microbe* 18 635–636. 10.1016/j.chom.2015.11.009 26651935

[B40] Gradel K. O. Nielsen H. L. Schonheyder H. C. Ejlertsen T. Kristensen B. Nielsen H. (2009). Increased short- and long-term risk of inflammatory bowel disease after *Salmonella* or campylobacter gastroenteritis. *Gastroenterology* 137 495–501. 10.1053/j.gastro.2009.04.001 19361507

[B41] Guan Y. Yang H. Han S. Feng L. Wang T. Ge J. (2017). Comparison of the gut microbiota composition between wild and captive sika deer (*Cervus nippon* hortulorum) from feces by high-throughput sequencing. *AMB Express* 7:212. 10.1186/s13568-017-0517-8 29170893 PMC5700909

[B42] Haas B. J. Gevers D. Earl A. M. Feldgarden M. Ward D. V. Giannoukos G. (2011). Chimeric 16S rrna sequence formation and detection in Sanger and 454-pyrosequenced Pcr amplicons. *Genome Res.* 21 494–504. 10.1101/gr.112730.110 21212162 PMC3044863

[B43] Hekmatdoost A. Feizabadi M. M. Djazayery A. Mirshafiey A. Eshraghian M. R. Yeganeh S. M. (2008). The effect of dietary oils on cecal microflora in experimental colitis in mice. *Indian J. Gastroenterol.* 27 186–189. 19112187

[B44] Houston F. Foster J. D. Chong A. Hunter N. Bostock C. J. (2000). Transmission of Bse by blood transfusion in sheep. *Lancet* 356 999–1000. 10.1016/s0140-6736(00)02719-7 11041403

[B45] Huang B. H. Chang C. W. Huang C. W. Gao J. Liao P. C. (2018). Composition and functional specialists of the gut microbiota of frogs reflect habitat differences and agricultural activity. *Front. Microbiol.* 8:2670. 10.3389/fmicb.2017.02670 29375532 PMC5768659

[B46] Huson D. H. Mitra S. Ruscheweyh H. J. Weber N. Schuster S. C. (2011). Integrative analysis of environmental sequences using Megan4. *Genome Res.* 21 1552–1560. 10.1101/gr.120618.111 21690186 PMC3166839

[B47] Ishaq S. L. Wright A.-D. G. (2012). Insight into the bacterial gut microbiome of the North American moose (*Alces alces*). *BMC Microbiol.* 12:212 10.1186/1471-2180-12-212 PMC3585231 22992344

[B48] IUCN SSC Antelope Specialist Group (2017). *Gazella subgutturosa, Goitered Gazelle. The Iucn Red List of Threatened Species* (Gland: IUCN), 2307–2385.

[B49] Karlsson F. H. Fak F. Nookaew I. Tremaroli V. Fagerberg B. Petranovic D. (2012). Symptomatic atherosclerosis is associated with an altered gut metagenome. *Nat. Commun.* 3:1245. 10.1038/ncomms2266 23212374 PMC3538954

[B50] Karlsson F. H. Tremaroli V. Nookaew I. Bergstrom G. Behre C. J. Fagerberg B. (2013). Gut metagenome in European women with normal, impaired and diabetic glucose control. *Nature* 498 99–103. 10.1038/nature12198 23719380

[B51] Khachatryan Z. A. Ktsoyan Z. A. Manukyan G. P. Kelly D. Ghazaryan K. A. Aminov R. I. (2008). Predominant role of host genetics in controlling the composition of gut microbiota. *PLoS One* 3:e3064. 10.1371/journal.pone.0003064 18725973 PMC2516932

[B52] Li H. Qu J. Li T. Wirth S. Zhang Y. Zhao X. (2018). Diet simplification selects for high gut microbial diversity and strong fermenting ability in high-altitude pikas. *Appl. Microbiol. Biotechnol.* 102 6739–6751. 10.1007/s00253-018-9097-z 29862448

[B53] Li J. Jia H. Cai X. Zhong H. Feng Q. Sunagawa S. (2014). An integrated catalog of reference genes in the human gut microbiome. *Nat. Biotechnol.* 32 834–841. 10.1038/nbt.2942 24997786

[B54] Li J. Lu Y. Fan C. Ding X. Qi K. (2015). Changes in gut microbiota in preschool obese children from two kindergartens in Beijing. *Chinese J. of Child Health Care* 23.

[B55] Li W. Godzik A. (2006). Cd-hit: a fast program for clustering and comparing large sets of protein or nucleotide sequences. *Bioinformatics* 22 1658–1659. 10.1093/bioinformatics/btl158 16731699

[B56] Li X. L. Gao J. Brierley G. Qiao Y. M. Zhang J. Yang Y. W. (2013). Rangeland degradation on the Qinghai-Tibet plateau: implications for rehabilitation. *Land Degrad. Dev.* 24 72–80. 10.1002/ldr.1108

[B57] Li Y. Hu X. Yang S. Zhou J. Zhang T. Qi L. (2017). Comparative analysis of the gut microbiota composition between captive and wild forest musk deer. *Front. Microbiol.* 8:1705. 10.3389/fmicb.2017.01705 28928728 PMC5591822

[B58] Lu A. (2016). Nutrient composition analysis of stipa purpurea in different areas of sanjiangyuan. *Heilongjiang Anim. Sci. Vet. Med.* 1 141–143. 10.13881/j.cnki.hljxmsy.2016.0043

[B59] Luo R. Liu B. Xie Y. Li Z. Huang W. Yuan J. (2012). Soapdenovo2:an empirically improved memory-efficient short-read de novo assembler. *Gigascience* 1 1–6. 23587118 10.1186/2047-217X-1-18 PMC3626529

[B60] Lyu T. Liu G. Zhang H. Wang L. Zhou S. Dou H. (2018). Changes in feeding habits promoted the differentiation of the composition and function of gut microbiotas between domestic dogs (*Canis lupus* familiaris) and gray wolves (*Canis lupus*). *AMB Express* 8:123. 10.1186/s13568-018-0652-x 30073560 PMC6072643

[B61] Ma Y. Ma S. Chang L. Wang H. Ga Q. Ma L. (2019). Gut microbiota adaptation to high altitude in indigenous animals. *Biochem. Biophys. Res. Commun.* 516 120–126. 10.1016/j.bbrc.2019.05.085 31196622

[B62] Magoč T. Salzberg S. L. (2011). Flash: fast length adjustment of short reads to improve genome assemblies. *Bioinformatics* 27 2957–2963. 10.1093/bioinformatics/btr507 21903629 PMC3198573

[B63] McDonald C. K. Macleod N. D. Lisson S. Corfield J. P. (2019). The Integrated Analysis Tool (Iat) – A model for the evaluation of crop-livestock and socio-economic interventions in smallholder farming systems. *Agric. Syst.* 176:102659 10.1016/j.agsy.2019.102659

[B64] McLellan S. L. Newton R. J. Vandewalle J. L. Shanks O. C. Huse S. M. Eren A. M. (2013). Sewage reflects the distribution of human faecal Lachnospiraceae. *Environ. Microbiol.* 15 2213–2227. 10.1111/1462-2920.12092 23438335 PMC4043349

[B65] Mende D. R. Waller A. S. Sunagawa S. Jarvelin A. I. Chan M. M. Arumugam M. (2012). Assessment of metagenomic assembly using simulated next generation sequencing data. *PLoS One* 7:e31386. 10.1371/journal.pone.0031386 22384016 PMC3285633

[B66] Menke S. Wasimuddin, Meier M. Melzheimer J. Mfune J. K. Heinrich S. (2014). Oligotyping reveals differences between gut microbiomes of free-ranging sympatric namibian carnivores (*Acinonyx jubatus*, *Canis mesomelas*) on a bacterial species-like level. *Front. Microbiol.* 5:526. 10.3389/fmicb.2014.00526 25352837 PMC4196554

[B67] Moeller A. H. Peeters M. Ndjango J. B. Li Y. Hahn B. H. Ochman H. (2013). Sympatric chimpanzees and gorillas harbor convergent gut microbial communities. *Genome Res.* 23 1715–1720. 10.1101/gr.154773.113 23804402 PMC3787267

[B68] Nam J. H. Yun Y. Kim H. S. Kim H. N. Jung H. J. Chang Y. (2018). Rosacea and its association with enteral microbiota in Korean females. *Exp. Dermatol.* 27 37–42. 10.1111/exd.13398 28636759

[B69] Nelson K. E. Zinder S. H. Hance I. Burr P. Odongo D. Wasawo D. (2003). Phylogenetic analysis of the microbial populations in the wild herbivore gastrointestinal tract: insights into an unexplored niche. *Environ. Microbiol.* 5 1212–1220. 10.1046/j.1462-2920.2003.00526.x 14641599

[B70] Nielsen H. B. Almeida M. Juncker A. S. Rasmussen S. Li J. Sunagawa S. (2014). Identification and assembly of genomes and genetic elements in complex metagenomic samples without using reference genomes. *Nat. Biotechnol.* 32 822–828. 10.1038/nbt.2939 24997787

[B71] Northwest Institute of Plateau Biology (1989). *Economic Animals in Qinghai Province.* Xining: Qinghai People’s Publishing House.

[B72] Oh J. Byrd A. L. Deming C. Conlan S. Program N. C. S. Kong H. H. (2014). Biogeography and individuality shape function in the human skin metagenome. *Nature* 514 59–64. 10.1038/nature13786 25279917 PMC4185404

[B73] Oksanen J. Blanchet F. G. Friendly M. Kindt R. Legendre P. Mcglinn D. (2019). *Community Ecology Package Version 2.5-6.*

[B74] Ostrowski S. Joseph W. (2006). Heterothermy of free-living Arabian sand gazelles (*Gazella subgutturosa* marica) in a desert environment. *J. Exp. Biol.* 209 1421–1429. 10.1242/jeb.02151 16574802

[B75] Ostrowski S. Mesochina P. Williams J. B. (2006). Physiological adjustments of sand Gazelles (*Gazella subgutturosa*) to a boom-or-bust economy: standard fasting metabolic rate, total evaporative water loss, and changes in the sizes of organs during food and water restriction. *Physiol. Biochem. Zool.* 79 810–819. 10.1086/504614 16826507

[B76] Payne A. N. Chassard C. Lacroix C. (2012). Gut microbial adaptation to dietary consumption of fructose, artificial sweeteners and sugar alcohols: implications for host-microbe interactions contributing to obesity. *Obes. Rev.* 13 799–809. 10.1111/j.1467-789X.2012.01009.x 22686435

[B77] Pereira-Marques J. Hout A. Ferreira R. M. Weber M. Pinto-Ribeiro I. Van Doorn L. J. (2019). Impact of host DNA and sequencing depth on the taxonomic resolution of whole metagenome sequencing for microbiome analysis. *Front. Microbiol.* 10:1277. 10.3389/fmicb.2019.01277 31244801 PMC6581681

[B78] Perry M. (2016). *Flexible Heatmaps for Functional Genomics and Sequence Feature S. R Package Version 1.11.0.*

[B79] Preidis G. A. Ajami N. J. Wong M. C. Bessard B. C. Conner M. E. Petrosino J. F. (2015). Composition and function of the undernourished neonatal mouse intestinal microbiome. *J. Nutr. Biochem.* 26 1050–1057. 10.1016/j.jnutbio.2015.04.010 26070414

[B80] Qin J. Li R. Raes J. Arumugam M. Burgdorf K. S. Manichanh C. (2010). A human gut microbial gene catalogue established by metagenomic sequencing. *Nature* 464 59–65. 10.1038/nature08821 20203603 PMC3779803

[B81] Qin J. Li Y. Cai Z. Li S. Zhu J. Zhang F. (2012). A metagenome-wide association study of gut microbiota in type 2 diabetes. *Nature* 490 55–60. 10.1038/nature11450 23023125

[B82] Qin N. Yang F. Li A. Prifti E. Chen Y. Shao L. (2014). Alterations of the human gut microbiome in liver cirrhosis. *Nature* 513 59–64. 10.1038/nature13568 25079328

[B83] Quan J. Cai G. Yang M. Zeng Z. Ding R. Wang X. (2019). Exploring the fecal microbial composition and metagenomic functional capacities associated with feed efficiency in commercial dly pigs. *Front. Microbiol.* 10:52. 10.3389/fmicb.2019.00052 30761104 PMC6361760

[B84] Quast C. Pruesse E. Yilmaz P. Gerken J. Schweer T. Yarza P. (2013). The Silva ribosomal Rna gene database project: improved data processing and web-based tools. *Nucleic Acids Res.* 41 D590–D596. 10.1093/nar/gks1219 23193283 PMC3531112

[B85] Ren T. Boutin S. Humphries M. M. Dantzer B. Gorrell J. C. Coltman D. W. (2017). Seasonal, spatial, and maternal effects on gut microbiome in wild red squirrels. *Microbiome* 5:163. 10.1186/s40168-017-0382-3 29268780 PMC5740981

[B86] Ross R. P. Mills S. Hill C. Fitzgerald G. F. Stanton C. (2010). Specific metabolite production by gut microbiota as a basis for probiotic function. *Int. Dairy J.* 20 269–276. 10.1016/j.idairyj.2009.12.003

[B87] Scasta J. D. Beck J. L. Angwin C. J. (2016). Meta-analysis of diet composition and potential conflict of wild horses with livestock and wild ungulates on Western Rangelands of North America. *Rangel. Ecol. Manag.* 69 310–318. 10.1016/j.rama.2016.01.001

[B88] Scher J. U. Sczesnak A. Longman R. S. Segata N. Ubeda C. Bielski C. (2013). Expansion of intestinal Prevotella copri correlates with enhanced susceptibility to arthritis. *eLife* 2:e01202. 10.7554/eLife.01202 24192039 PMC3816614

[B89] Schloss P. D. Westcott S. L. Ryabin T. Hall J. R. Hartmann M. Hollister E. B. (2009). Introducing mothur: open-source, platform-independent, community-supported software for describing and comparing microbial communities. *Appl. Environ. Microbiol.* 75 7537–7541. 10.1128/Aem.01541-09 19801464 PMC2786419

[B90] Scupham A. J. Patton T. G. Bent E. Bayles D. O. (2008). Comparison of the cecal microbiota of domestic and Wild Turkeys. *Microb. Ecol.* 56 322–331. 10.1007/s00248-007-9349-4 18183454

[B91] Seekatz A. M. Panda A. Rasko D. A. Toapanta F. R. Eloe-Fadrosh E. A. Khan A. Q. (2013). Differential response of the cynomolgus macaque gut microbiota to *Shigella* infection. *PLoS One* 8:e64212. 10.1371/journal.pone.0064212 23755118 PMC3673915

[B92] Segata N. Izard J. Waldron L. Gevers D. Miropolsky L. Garrett W. S. (2011). Metagenomic biomarker discovery and explanation. *Genome Biol.* 12 1–18. 10.1186/gb-2011-12-6-r60 21702898 PMC3218848

[B93] Shen D. (2012). Gut microbial ecosystem and obesity. *Chin. J. Microecol.* 24 91–93. 10.13381/j.cnki.cjm.2012.01.027

[B94] Shi X. Zhao Y. Dai S. Xu L. Li Y. Jia H. (2005). Research on climate change of Qaidam Basin since 1961. *J. Desert Res.* 25 123–128.

[B95] Singh B. Chaudhary L. C. Agarwal N. Kamra D. N. (2011). Phenotypic and phylogentic characterisation of tannin degrading/tolerating bacterial isolates from the rumen of goats fed on pakar (*Ficus infectoria*)leaves. *J. Appl. Anim. Res.* 39 120–124. 10.1080/09712119.2011.558682

[B96] Skirrow M. B. (1994). Diseases due to campylobacter, *Helicobacter* and related bacteria. *J. Comp. Path.* 111 113–149. 10.1016/s0021-9975(05)80046-5 7806700

[B97] Smith C. C. Snowberg L. K. Gregory Caporaso J. Knight R. Bolnick D. I. (2015). Dietary input of microbes and host genetic variation shape among-population differences in stickleback gut microbiota. *ISME J.* 9 2515–2526. 10.1038/ismej.2015.64 25909977 PMC4611514

[B98] Sommer F. Stahlman M. Ilkayeva O. Arnemo J. M. Kindberg J. Josefsson J. (2016). The gut microbiota modulates energy metabolism in the hibernating brown Bear *Ursus arctos*. *Cell Rep.* 14 1655–1661. 10.1016/j.celrep.2016.01.026 26854221

[B99] Sullam K. E. Essinger S. D. Lozupone C. A. O’connor M. P. Rosen G. L. Knight R. (2012). Environmental and ecological factors that shape the gut bacterial communities of fish: a meta-analysis. *Mol. Ecol.* 21 3363–3378. 10.1111/j.1365-294X.2012.05552.x 22486918 PMC3882143

[B100] Sun B. Wang X. Bernstein S. Huffman M. A. Xia D. P. Gu Z. (2016). Marked variation between winter and spring gut microbiota in free-ranging Tibetan Macaques (*Macaca thibetana*). *Sci. Rep.* 6:26035. 10.1038/srep26035 27180722 PMC4867428

[B101] Sunagawa S. Coelho L. P. Chaffron S. Kultima J. R. Labadie K. Salazar G. (2015). Structure and function of the global ocean microbiome. *Science* 348:1261359. 10.1126/science.1261359 25999513

[B102] Supratman H. Ramdani D. Kuswaryan S. Budinuryanto D. C. Joni I. M. (2018). Application of probiotics and different size of sodium bicarbonate powders for feedlot sheep fattening. *AIP Conf. Proc.* 1927:030045 10.1063/1.5021238

[B103] Tanca A. Fraumene C. Manghina V. Palomba A. Abbondio M. Deligios M. (2017). Diversity and functions of the sheep faecal microbiota: a multi-omic characterization. *Microb. Biotechnol.* 10 541–554. 10.1111/1751-7915.12462 28165194 PMC5404191

[B104] Thomas N. A. Olvera-Ramirez A. M. Abecia L. Adam C. L. Edwards J. E. Cox G. F. (2019). Characterisation of the effect of day length, and associated differences in dietary intake, on the gut microbiota of Soay sheep. *Arch. Microbiol.* 201 889–896. 10.1007/s00203-019-01652-w 30968220 PMC6687699

[B105] Tremaroli V. Bäckhed F. (2012). Functional interactions between the gut microbiota and host metabolism. *Nature* 489 242–249. 10.1038/nature11552 22972297

[B106] Trompette A. Gollwitzer E. S. Yadava K. Sichelstiel A. K. Sprenger N. Ngom-Bru C. (2014). Gut microbiota metabolism of dietary fiber influences allergic airway disease and hematopoiesis. *Nat. Med.* 20 159–166. 10.1038/nm.3444 24390308

[B107] Turpin W. Espin-Garcia O. Xu W. Silverberg M. S. Kevans D. Smith M. I. (2016). Association of host genome with intestinal microbial composition in a large healthy cohort. *Nat. Genet.* 48 1413–1417. 10.1038/ng.3693 27694960

[B108] Vrieze A. Holleman F. Zoetendal E. G. De Vos W. M. Hoekstra J. B. Nieuwdorp M. (2010). The environment within: how gut microbiota may influence metabolism and body composition. *Diabetologia* 53 606–613. 10.1007/s00125-010-1662-7 20101384 PMC2830587

[B109] Wang H. Wu D. (2018). Study on the nutrition composition and feeding value of phragmites before and after ensiling. *Cereal Feed Ind.* 2 59–61.

[B110] Wang J. Fan H. Han Y. Zhao J. Zhou Z. (2017). Characterization of the microbial communities along the gastrointestinal tract of sheep by 454 pyrosequencing analysis. *Asian Australas. J. Anim. Sci.* 30 100–110. 10.5713/ajas.16.0166 27383798 PMC5205584

[B111] Wang Q. Garrity G. M. Tiedje J. M. James R. C. (2007). Naive Bayesian classifier for rapid assignment of rrna sequences into the new bacterial taxonomy. *Appl. Environ. Microbiol.* 73 5261–5267. 10.1128/Aem.00062-07 17586664 PMC1950982

[B112] Wang X. Xiao H. Chen G. Zhao X. Huang C. Chen C. (2011). Isolation of high-quality RNA from *Reaumuria soongorica*, a desert plant rich in secondary metabolites. *Mol. Biotechnol.* 48 165–172. 10.1007/s12033-010-9357-3 21136208

[B113] Wei L. Bai S. Li J. Hou X. Wang X. Li H. (2014). QTL positioning of thousand wheat grain weight in Qaidam Basin. *Open J. Genet.* 04 239–244. 10.4236/ojgen.2014.43024

[B114] Wickham H. Chang W. Henry L. Pedersen T. L. Takahashi K. Wilke C. (2019). *Create Elegant Data Visualisations Using the Grammar of Graphics ggplot2 Version 3.2.1.*

[B115] Woodcock B. A. Pywell R. F. Roy D. B. Rose R. J. Bell D. (2005). Grazing management of calcareous grasslands and its implications for the conservation of beetle communities. *Biol. Conserv.* 125 193–202. 10.1016/j.biocon.2005.03.017

[B116] Wu Z. Deng J. Tian Y. Wang X. Yuan Y. Wang J. (2017). Anlysis and evaluation forage quality of four Nitraria Species. *J. Gansu Agric. Univ.* 52 97–100. 10.13432/j.cnki.jgsau.2017.06.016

[B117] Xu B. Xu W. Li J. Dai L. Xiong C. Tang X. (2015). Metagenomic analysis of the Rhinopithecus bieti fecal microbiome reveals a broad diversity of bacterial and glycoside hydrolase profiles related to lignocellulose degradation. *BMC Genomics* 16:174. 10.1186/s12864-015-1378-7 25887697 PMC4369366

[B118] Xu W. Fang Q. Liu W. Yang W. (2008a). Food habits of goitered gazelles (*Gazella subgutturosa* sairensis) in northern Xinjiang. *Acta Theriol. Sin.* 28 280–286.

[B119] Xu W. Qiao J. Liu W. Yang W. (2008b). Ecology and biology of *Gazella subgutturosa*: current situation of studies. *Chin. J. Ecol.* 27 257–262.

[B120] Xu X. Xu P. Ma C. Tang J. Zhang X. (2013). Gut microbiota, host health, and polysaccharides. *Biotechnol. Adv.* 31 318–337. 10.1016/j.biotechadv.2012.12.009 23280014

[B121] Xue D. Huai C. Fang C. Yixin H. Chuan Z. Dan Z. (2016). Analysis of the rumen bacteria and methanogenic archaea of yak (Bos grunniens) steers grazing on the Qinghai-Tibetan Plateau. *Livest. Sci.* 188 61–71. 10.1016/j.livsci.2016.04.009

[B122] Yang D. Qi Y. Wang X. Wang F. Wu J. (2019). Ceratoides and alfalfa added weight of sunit sheep and influence their rumen bacteria. *Chin. J. Anim. Sci.* 55 87–91. 10.19556/j.0258-7033.2019-06-087

[B123] Yang Y. Deng Y. Cao L. (2016). Characterising the interspecific variations and convergence of gut microbiota in Anseriformes herbivores at wintering areas. *Sci. Rep.* 6:32655. 10.1038/srep32655 27600170 PMC5013396

[B124] Zeller G. Tap J. Voigt A. Y. Sunagawa S. Kultima J. R. Costea P. I. (2014). Potential of fecal microbiota for early-stage detection of colorectal cancer. *Mol. Syst. Biol.* 10:766. 10.15252/msb.20145645 25432777 PMC4299606

[B125] Zhang H. Shao M. Huang H. Wang S. Ma L. Wang H. (2018). The dynamic distribution of small-tail han sheep microbiota across different intestinal segments. *Front. Microbiol.* 9:32. 10.3389/fmicb.2018.00032 29445360 PMC5797768

[B126] Zhang H. Sparks J. B. Karyala S. V. Settlage R. Luo X. M. (2015). Host adaptive immunity alters gut microbiota. *ISME J.* 9 770–781. 10.1038/ismej.2014.165 25216087 PMC4331585

[B127] Zhao F. Huang Z. Zhou G. Li H. Xu X. Li C. (2017). Dietary proteins rapidly altered the microbial composition in rat caecum. *Curr. Microbiol.* 74 1447–1452. 10.1007/s00284-017-1339-2 28831546

[B128] Zhao X. Ren B. Li D. Garber P. A. Zhu P. Xiang Z. (2019). Climate change, grazing, and collecting accelerate habitat contraction in an endangered primate. *Biol. Conserv.* 231 88–97. 10.1016/j.biocon.2019.01.007

[B129] Zhong Z. Zhou G. Yang L. Liu H. Song W. (2014). The biomass allocation patterns of desert shrub vegetation in the Qiadam Basin, Qinghai, China. *J. Desert Res.* 34 1042–1048.

[B130] Zhu W. Lomsadze A. Borodovsky M. (2010). Ab initio gene identification in metagenomic sequences. *Nucleic Acids Res.* 38:e132. 10.1093/nar/gkq275 20403810 PMC2896542

[B131] Zmora N. Suez J. Elinav E. (2018). You are what you eat: diet, health and the gut microbiota. *Nat. Rev. Gastroenterol. Hepatol.* 16 35–56. 10.1038/s41575-018-0061-2 30262901

