# Supplementary material for: Gut Microbiota Plasticity Influences the Adaptability of Wild and Domestic Animals in Co-inhabited Areas
Source: Front Microbiol. 2020 Feb 7;11:125. doi: 10.3389/fmicb.2020.00125 (PMC7018712; doi:10.3389/fmicb.2020.00125)
Supplement: Supplementary file 1 [file Data_Sheet_1.PDF]

Supplementary

Supplementary Table A: In raw data, the quality information of every sample. # means no calculation.

Sample name	Total reads	Combined reads	Uncombined reads	Percent combined(%)	Min len(bp)	Max len(bp)	Avg len(bp)
WGS1	74,721	67,761	6,960	90.69	64	441	409
WGS2	74,855	66,465	8,390	88.79	287	441	410
WGS3	87,181	77,750	9,431	89.18	245	441	407
WGS4	76,548	68,887	7,661	89.99	295	438	410
WGS5	84,352	76,964	7,388	91.24	246	431	409
WGS6	83,442	75,925	7,517	90.99	307	437	408
WGS7	70,324	63,645	6,679	90.50	247	431	408
WGS8	73,770	67,106	6,664	90.97	228	441	407
WGS9	88,754	78,801	9,953	88.79	248	441	410
WGS10	65,855	58,823	7,032	89.32	248	433	409
WGS11	89,070	80,335	8,735	90.19	59	441	409
WGS12	86,496	78,928	7,568	91.25	270	434	409
WGS13	57,354	51,194	6,160	89.26	154	440	409
WGS14	80,045	71,274	8,771	89.04	247	441	409
WGS15	89,290	79,337	9,953	88.85	254	440	410
WGS16	75,577	68,300	7,277	90.37	300	432	410
WGS17	88,183	77,725	10,458	88.14	269	441	410
WGS18	76,436	67,933	8,503	88.88	242	441	410
WGS19	60,057	53,723	6,334	89.45	263	441	409
WGS20	79,810	70,986	8,824	88.94	64	436	411
WSE10	81,523	72,468	9,055	88.89	300	441	412
WSE11	71,913	65,204	6,709	90.67	232	441	411
WSE12	84,335	74,541	9,794	88.39	322	441	411
WSE13	84,245	76,183	8,062	90.43	315	441	410
WSE1	89,750	79,956	9,794	89.09	265	441	411
WSE2	73,327	64,759	8,568	88.32	284	441	412
WSE3	75,816	68,241	7,575	90.01	269	441	412
WSE4	83,251	75,220	8,031	90.35	64	435	411
WSE5	71,843	70,358	1,485	97.93	305	441	419
WSE6	85,035	74,846	10,189	88.02	243	441	411
WSE7	84,762	74,926	9,836	88.40	64	441	412
WSE8	89,512	80,659	8,853	90.11	287	440	410
WSE9	88,889	79,694	9,195	89.66	249	435	410
**Average Number**	79,585	71,482	8,103	89.85	234.42	438.85	410.15
**Total Number**	2,626,321	2,358,917	267,404	2,965	#	#	#

Supplementary Table B: In clean data, the quality information of every sample. # means no calculation.

Sample Name	Raw PE reads Number	Qualified reads number	AvgLen(nt)	Q20	Q30	GC%	Effective%
WGS1	74721	61069	409	98.28	96.62	52.99	78.63
WGS2	74855	59543	409	98.09	96.20	53.05	76.88
WGS3	87181	70192	407	98.23	96.51	53.5	77.88
WGS4	76548	61560	410	98.21	96.50	53.15	76.82
WGS5	84352	69641	408	98.29	96.64	52.95	79.75
WGS6	83442	68800	408	98.27	96.61	52.98	79.28
WGS7	70324	57432	408	98.29	96.63	53.00	78.15
WGS8	73770	60434	407	98.27	96.60	53.42	79.51
WGS9	88754	70422	410	98.13	96.29	52.81	77.48
WGS10	65855	52908	408	98.15	96.32	53.20	75.85
WGS11	89070	72133	409	98.27	96.60	52.99	77.75
WGS12	86496	71024	409	98.32	96.70	52.71	79.55
WGS13	57354	45828	409	98.12	96.28	52.94	76.90
WGS14	80045	63974	409	98.19	96.39	52.97	77.18
WGS15	89290	70914	409	98.14	96.29	52.77	76.15
WGS16	75577	61243	410	98.27	96.60	52.86	77.80
WGS17	88183	69286	410	98.17	96.37	52.85	75.83
WGS18	76436	60614	409	98.14	96.30	52.91	76.90
WGS19	60057	48108	409	98.14	96.31	52.99	77.50
WGS20	79810	63417	411	98.18	96.36	52.60	76.88
WSE1	89750	71678	411	98.23	96.47	52.68	77.75
WSE2	73327	57288	412	98.09	96.21	52.62	76.09
WSE3	75816	60684	411	98.22	96.52	52.83	77.67
WSE4	83251	67323	410	98.25	96.58	52.39	78.55
WSE5	71843	57063	415	98.18	96.42	53.30	60.47
WSE6	85035	66490	411	98.12	96.25	52.92	76.15
WSE7	84762	66379	412	98.11	96.25	52.78	76.12
WSE8	89512	72205	409	98.26	96.59	52.94	78.68
WSE9	88889	71774	410	98.19	96.40	52.76	79.05
WSE10	81523	64584	411	98.11	96.26	52.87	77.65
WSE11	71913	58619	411	98.26	96.57	53.04	79.97
WSE12	84335	66382	411	98.15	96.33	52.85	76.98
WSE13	84245	68360	409	98.24	96.55	52.59	79.45
**Total Number**	2626321	2107371	13521	#	#	#	#
**Average Number**	79585.48485	63859.72727	409.72	98.19879	96.44	52.91545	77.18939

Supplementary Table C: The functions in Metabolism, the relative abundance and *p* values of 49 functions of gut microbiota showed significant differences between groups of goitered gazelle and sheep in co-inhabited area based on KEGG database.

Functions at third level	Goitered gazelle	Sheep	*p* value
Starch and sucrose metabolism	0.0086%	0.0039%	0.0073
Cysteine and methionine metabolism	0.0080%	0.0034%	0.0108
Galactose metabolism	0.0063%	0.0026%	0.0031
Peptidoglycan biosynthesis	0.0058%	0.0028%	0.0219
Oxidative phosphorylation	0.0056%	0.0030%	0.0336
Phenylalanine, tyrosine and tryptophan biosynthesis	0.0056%	0.0016%	0.0016
Lysine biosynthesis	0.0042%	0.0018%	0.0082
Arginine biosynthesis	0.0035%	0.0019%	0.0386
Photosynthesis	0.0032%	0.0016%	0.0200
Porphyrin and chlorophyll metabolism	0.0031%	0.0011%	0.0110
Thiamine metabolism	0.0031%	0.0014%	0.0130
Terpenoid backbone biosynthesis	0.0028%	0.0007%	0.0006
Pantothenate and CoA biosynthesis	0.0028%	0.0013%	0.0145
Valine, leucine and isoleucine biosynthesis	0.0025%	0.0011%	0.0115
Fatty acid biosynthesis	0.0025%	0.0008%	0.0023
Histidine metabolism	0.0024%	0.0010%	0.0056
Cyanoamino acid metabolism	0.0022%	0.0011%	0.0156
Nicotinate and nicotinamide metabolism	0.0019%	0.0009%	0.0406
Streptomycin biosynthesis	0.0019%	0.0008%	0.0107
Nitrogen metabolism	0.0018%	0.0010%	0.0455
Glycerolipid metabolism	0.0018%	0.0009%	0.0134
Phenylalanine metabolism	0.0016%	0.0006%	0.0058
Monobactam biosynthesis	0.0015%	0.0006%	0.0124
Phenylpropanoid biosynthesis	0.0014%	0.0004%	0.0008
Fatty acid degradation	0.0014%	0.0007%	0.0495
Folate biosynthesis	0.0013%	0.0003%	0.0030
Biotin metabolism	0.0012%	0.0002%	0.0001

D-Glutamine and D-glutamate metabolism	0.0012%	0.0004%	0.0020
Sulfur metabolism	0.0012%	0.0004%	0.0076
Novobiocin biosynthesis	0.0010%	0.0004%	0.0046
Ascorbate and aldarate metabolism	0.0010%	0.0004%	0.0239
Vitamin B6 metabolism	0.0009%	0.0004%	0.0105
C5-Branched dibasic acid metabolism	0.0009%	0.0003%	0.0061
Biosynthesis of ansamycins	0.0008%	0.0004%	0.0419
Polyketide sugar unit biosynthesis	0.0008%	0.0003%	0.0132
Prodigiosin biosynthesis	0.0007%	0.0002%	0.0004
Acarbose and validamycin biosynthesis	0.0006%	0.0003%	0.0197
D-Alanine metabolism	0.0005%	0.0002%	0.0210
Biosynthesis of unsaturated fatty acids	0.0005%	0.0001%	0.0017
Benzoate degradation	0.0005%	0.0001%	0.0111
Phosphonate and phosphinate metabolism	0.0002%	0.0001%	0.0120
Synthesis and degradation of ketone bodies	0.0002%	0.0000%	0.0091
D-Arginine and D-ornithine metabolism	0.0002%	0.0000%	0.0283
Phenazine biosynthesis	0.0002%	0.0000%	0.0007
Primary bile acid biosynthesis	0.0001%	0.0000%	0.0085
Xylene degradation	0.0001%	0.0000%	0
Arachidonic acid metabolism	0.0001%	0.0000%	0.0391
Dioxin degradation	0.0001%	0.0000%	0.0032
Lipoic acid metabolism	0.0001%	0.0000%	0.0038

Supplementary Table D: The functions in Cellular Processes, the relative abundance and *p* values of 6 functions of gut microbiota showed significant differences between groups of goitered gazelle and sheep in co-inhabited area based on KEGG database.

Functions at third level	Goitered gazelle	Sheep	*p* value
Biofilm formation - Pseudomonas aeruginosa	0.000199%	0.000042%	0.0050
Ferroptosis	0.000845%	0.000351%	0.0075
Cell cycle - Caulobacter	0.004082%	0.001868%	0.0100
Biofilm formation - Vibrio cholerae	0.001721%	0.000712%	0.0153
Autophagy - yeast	0.000013%	0.000000%	0.0325

Peroxisome	0.001497%	0.000805%	0.0433
